# Supplementary material for: HSPA2 Chaperone Contributes to the Maintenance of Epithelial Phenotype of Human Bronchial Epithelial Cells but Has Non-Essential Role in Supporting Malignant Features of Non-Small Cell Lung Carcinoma, MCF7, and HeLa Cancer Cells
Source: Cancers (Basel). 2020 Sep 24;12(10):2749. doi: 10.3390/cancers12102749 (PMC7598654; doi:10.3390/cancers12102749)
Supplement: Supplementary file 1 [file cancers-12-02749-s001.zip › cancers-899297 supplementary materials.docx]

Supplementary Materials:

HSPA2 Chaperone Contributes to the Maintenance of Epithelial Phenotype of Human Bronchial Epithelial Cells but Has Non-Essential Role in Supporting Malignant Features of Non-Small Cell Lung Carcinoma, MCF7, and HeLa Cancer Cells

Damian Robert Sojka, Agnieszka Gogler-Pigłowska, Katarzyna Klarzyńska, Marta Klimczak, Alicja Zylicz, Magdalena Głowala-Kosińska, Zdzisław Krawczyk and Dorota Scieglinska


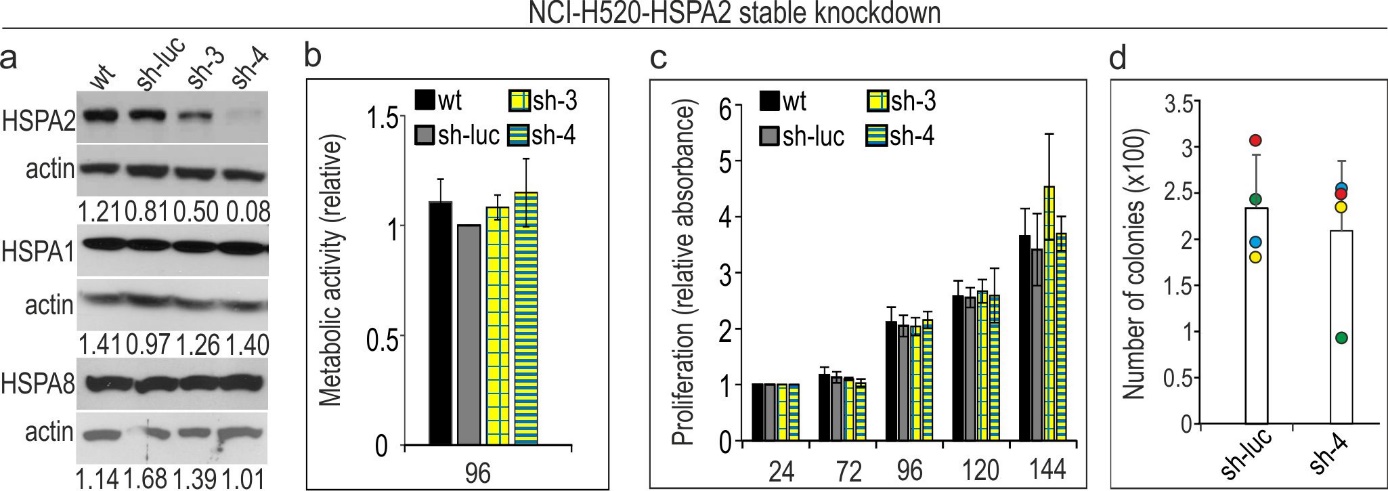


**Figure S1.** Effects of the *HSPA2* knockdown mediated by lentiviral vectors on the growth of NCI-H520 cells. **a)** Representative immunoblots showing HSPA2, HSPA1 and HSPA8 protein levels in wild-type (wt), control (sh-luc) and HSPA2-deficient (sh-3, sh-4) cells. actin - a protein loading control. Control sh-luc cells were stably transduced with the non-targeting shRNAluc sequence; sh-3 and sh-4 cells were transduced with the *HSPA2*-targeting shRNA3 or shRNA4 sequences, respectively The numbers below each lane represent the protein ratio normalized to the actin level. **b)** Cell metabolic activity was measured using the MTS assay after 96 hours of cell growth. Results are expressed as mean ± SD (n = 6, each in three technical replicas) in relation to sh-luc cells. **c)** The cell proliferation rate was examined using the crystal violet staining assay (n=2, each in six technical replicas). Results are representative of two independent experiments; and are expressed as mean ± SD (six technical replicas) in relation to values obtained at 24 h after plating. **d**) Results of the clonogenic assay. Cells were plated onto 6-well dishes (4 × 10^3^ cells / well) and cultured for 15-17 days). The colonies were counted manually (mean ± SD, n = 4, each in 3 technical replicas).

**
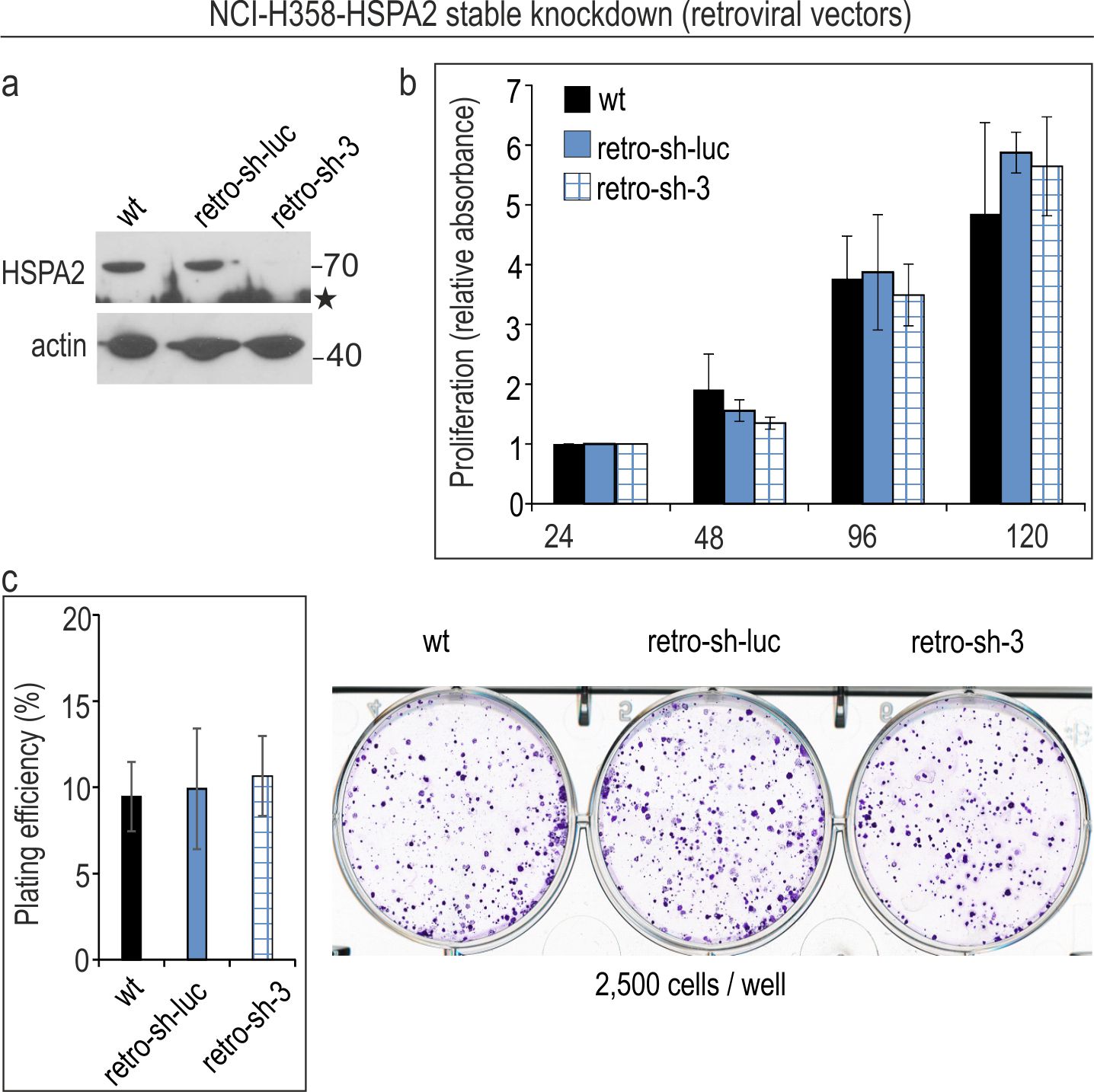
**

**Figure S2.** Effects of the *HSPA2* knockdown mediated by retroviral vectors on the growth of NCI-H358 cells. **a)** Representative immunoblots showing the protein levels of HSPA2 in wild-type (wt), control (retro-sh-luc) and HSPA2-deficient (retro-sh-3) cells (molecular weight in kDa is indicated). actin - a protein loading control. The cell line (retro-sh-3) with stable knockdown of the *HSPA2* gene expression was established using a retroviral vector encoding the *HSPA2*-targeting shRNA3 sequences. shRNA sequences (shRNA3 or non-targeting shRNAluc) were cloned into the pSIREN-RetroQ plasmid (Clontech) and transfected into the RetroPack PT67 (Clontech) packaging cells to produce retroviruses. NCI-H358 cells were incubated with the retroviral supernatants for 24 h, transduction was repeated in triplicate in 12 h intervals. Transduced cells were selected using puromycin. The asterisk indicates non-specific bands. **b)** Cell proliferation rate was examined using the crystal violet staining assay. Cells (6x10^3^ / well) were plated into 24 –well plates and cultured for up to 120 h. Results are mean ± SD (n = 2, each in six technical replicas) in relation to values obtained at 24 h after plating. **d**) Results of the clonogenic assay. Cells were plated onto 6-well dishes and cultured for 12 days). The colonies were counted manually (mean ± SD, n = 2, each in 3 technical replicas).


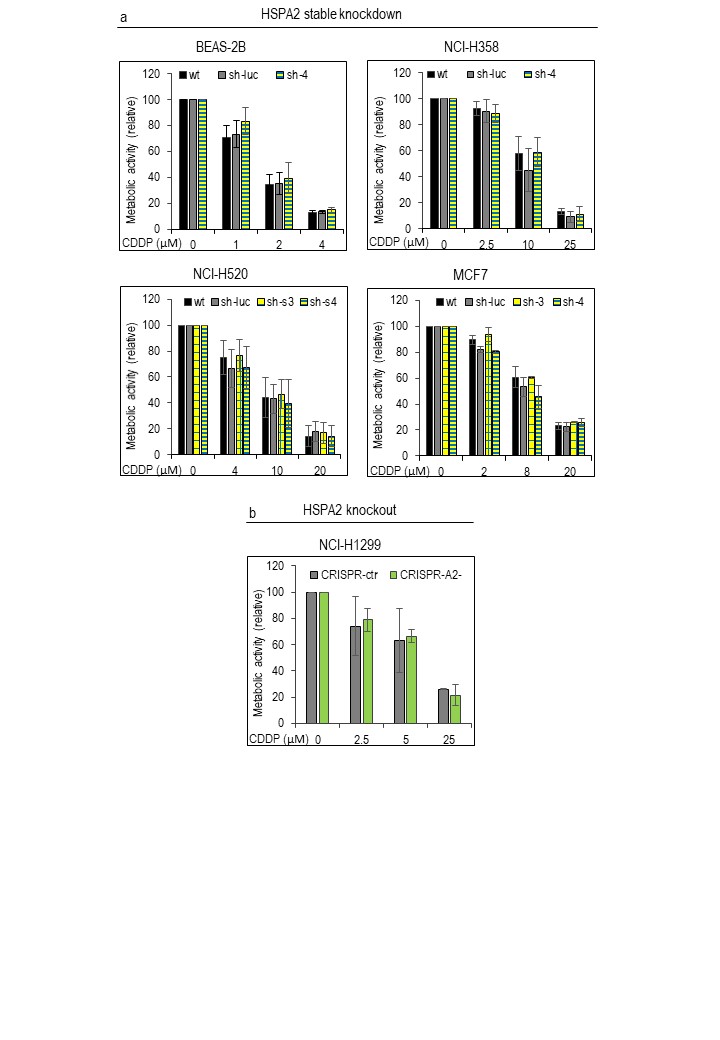


**Figure S3.** Decreased level of HSPA2 has no effect on resistance of human bronchial epithelial and cancer cells to cisplatin (CDDP). (a-b) Cells (5 × 10^3^, BEAS-2B; 8 × 10^3^, NCI-H358; 15 × 10^3^, NCI-H520; 4 × 10^3^, MCF7; 3 × 10^3^, NCI-H1299 per well) were seeded into 96-well plates and incubated with the drug for 72 h. Control sh-luc cells were stably transduced with the non-targeting shRNAluc sequence; sh-3 and sh-4 cells were transduced with the HSPA2-targeting shRNA3 or shRNA4 sequences. CRISPR-ctr cells transfected with CRISPR/Cas9 control plasmid; CRISPR-A2- cells transfected with the *HSPA2* gene editing CRISPR/Cas9 plasmids. Cell viability was measured using the MTS assay (CellTiter 96 Aqueous One Solution Assay; Promega; Madison, WI, USA) according to a manufacturer’s protocol. Results are expressed relative to the untreated control (mean ± SD; n = 2, BEAS-2B, MCF7; n ≥ 3, NCI-H358, NCI-H520, NCI-H1299, each in three technical repeats).


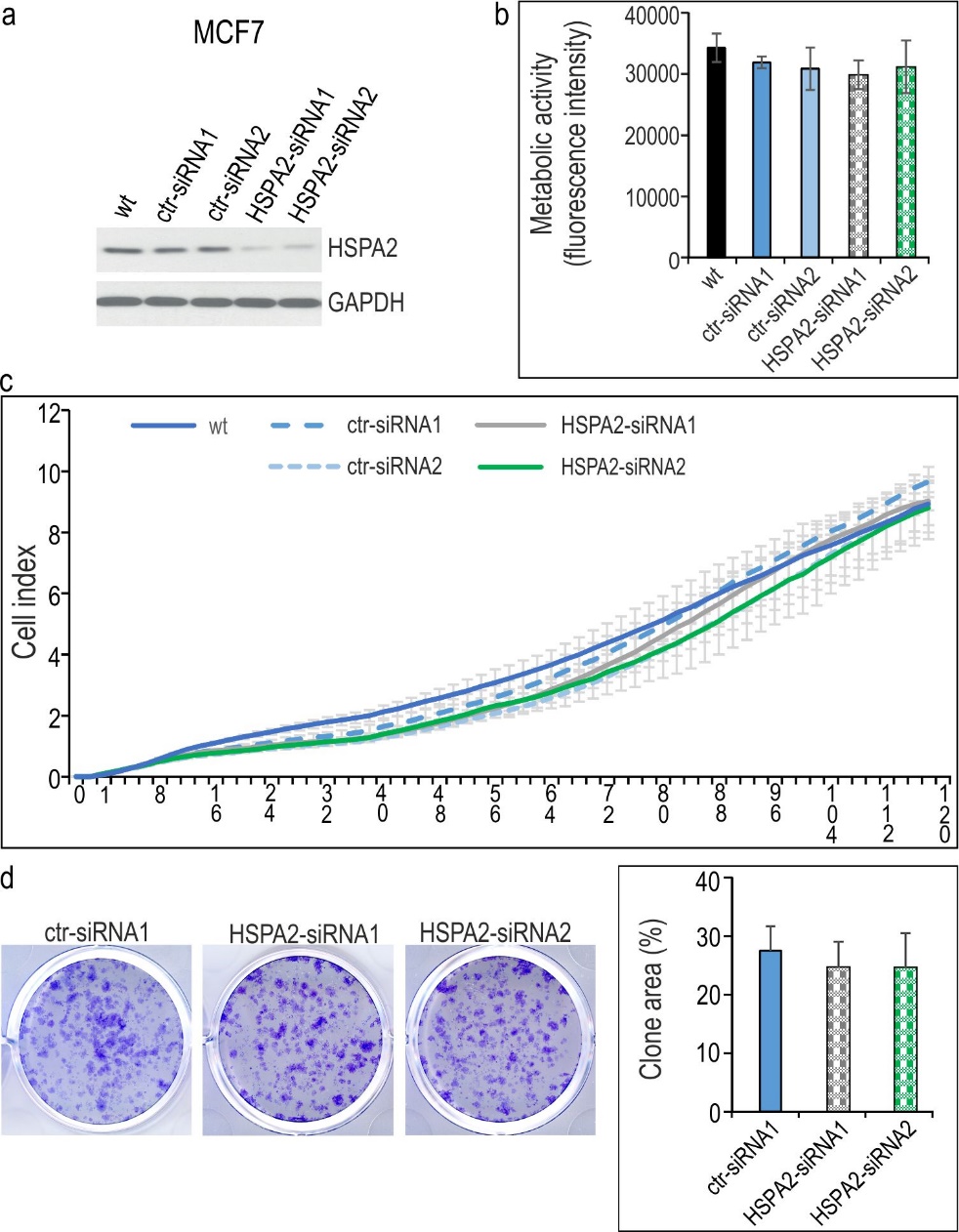


**Figure S4.** siRNA-mediated silencing of the *HSPA2* gene expression has no impact on the growth of breast carcinoma MCF7 cells. **a)** Representative immunoblots showing the HSPA2 protein level in wild-type (wt), control (ctr-siRNA1, ctr-siRNA2) and HSPA2-deficient (HSPA2-siRNA1, HSPA2-siRNA2) cells. GAPDH - a protein loading control. Cells were transfected with the scrambled ctr-siRNA1 (ThermoFisher Scientific cat. no. 4390843) or ctr-siRNA2 (ThermoFisher Scientific cat. no. 4390846) sequences; HSPA2-siRNA1 and HSPA2-siRNA2 - cells transfected with the *HSPA2*-targeting siRNA1 (ThermoFisher Scientific cat. No. s6974) or siRNA2 (ThermoFisher Scientific cat. No. s6973) sequences, respectively. The transfections with 40 nM siRNA were performed with GenMute siRNA Transfection Reagent (SignaGen Labolatories, Frederic, MD, USA) according to manufacturer’s instructions. For efficient knockdown, transfection was repeated after 24 h. **b)** Metabolic activity assessed using the resazurin reduction assay. Results are expressed as mean ± SD (n = 3). For resazurin assay MCF7 cells, 24 h post second siRNA transfection, were seeded in 96-well plates (1 x 10^4^ / well). Cells were cultured for 72 h, resazurin (Sigma Aldrich) was added to cell culture medium to a final concentration of 15 μg/ml and incubated with cells for 7 h at 37°C. Fluorescence of metabolized resazurin was measured at 560 nm excitation and 590 nm emission wavelengths using microplate reader (Infinite M1000, Tecan). **c)** Real-time analysis of cells growth was examined using xCELLigence system. 24 h post second siRNA transfection, MCF7 cells were seeded (1.5 x 10^4^ / well) in each well of E-plate 16 (ACEA Biosciences) and placed in the xCELLigence RTCA DP Instrument (ACEA Biosciences) in an incubator. After reaching plateau, cells were treated with doxorubicin (1 μM) and further monitored. **d)** Results of the colony formation assay (mean ± SD, n = 3).
