# Supplementary material for: HSPA2 Chaperone Contributes to the Maintenance of Epithelial Phenotype of Human Bronchial Epithelial Cells but Has Non-Essential Role in Supporting Malignant Features of Non-Small Cell Lung Carcinoma, MCF7, and HeLa Cancer Cells
Source: Cancers (Basel). 2020 Sep 24;12(10):2749. doi: 10.3390/cancers12102749 (PMC7598654; doi:10.3390/cancers12102749)
Supplement: Supplementary file 1 [file cancers-12-02749-s001.zip › cancers-899297 uncropped figures.docx]

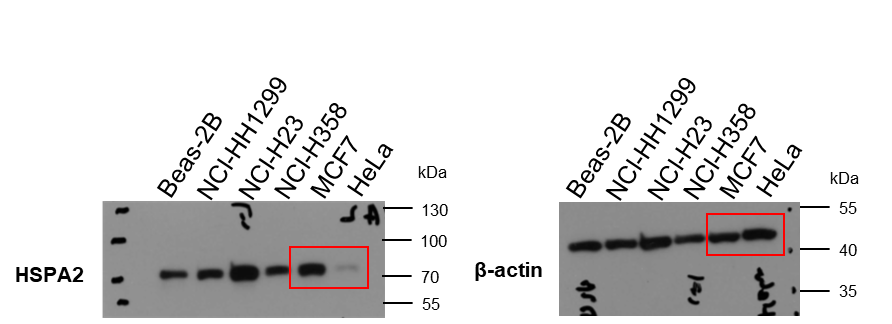


**
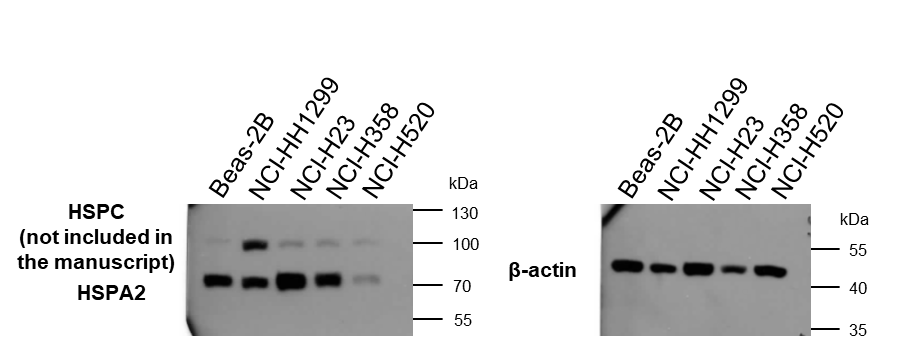
**

**Figure S5.** Unprocessed original scans of autoradiograms (immunoblots) included in Fig. 1a

**
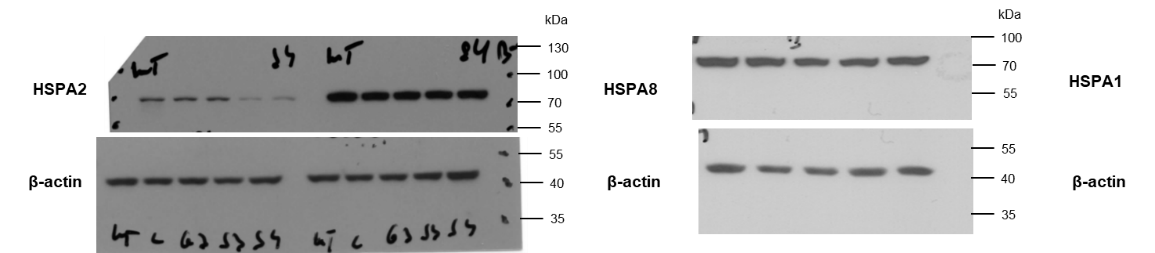
**

**Figure S6.** Unprocessed original scans of autoradiograms (immunoblots) included in Fig. 2a


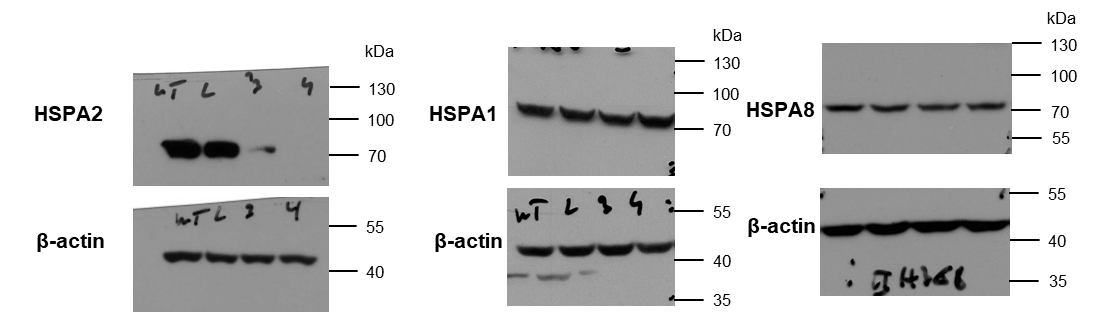


**Figure S7.** Unprocessed original scans of autoradiograms (immunoblots) included in Fig. 3a


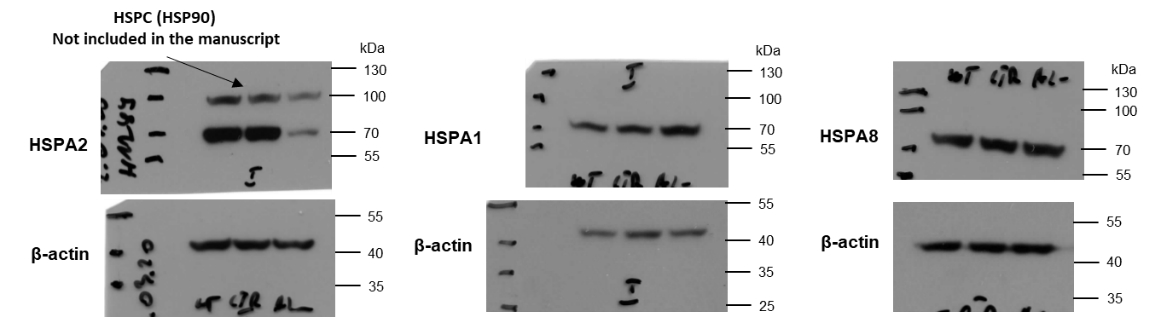


**Figure S8.** Unprocessed original scans of autoradiograms (immunoblots) included in Fig. 3e


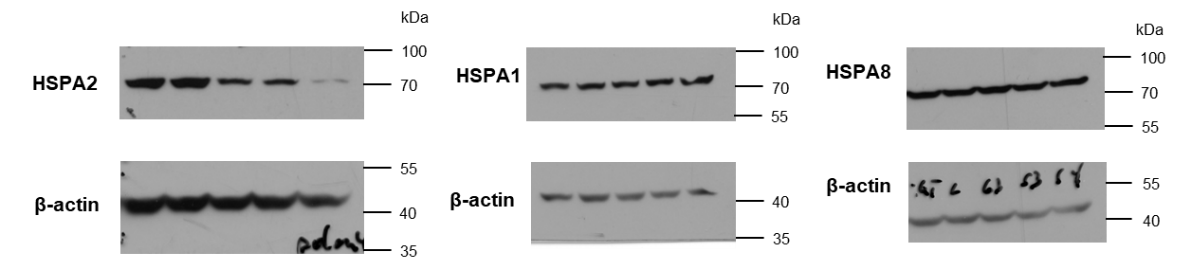


**Figure S9.** Unprocessed original scans of autoradiograms (immunoblots) included in Fig. 6a

**
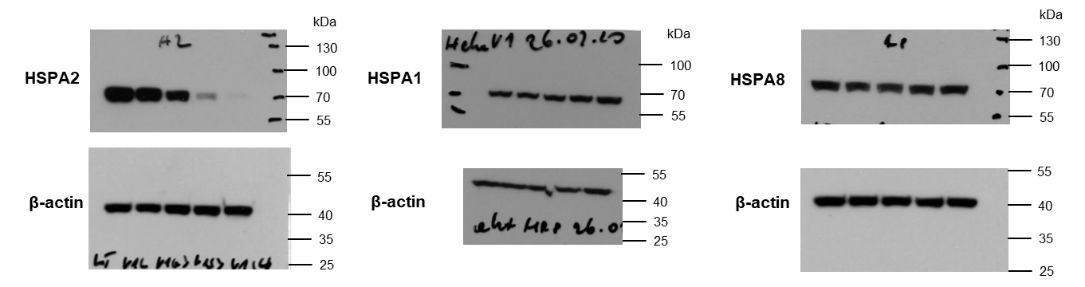
**

**Figure S10.** Unprocessed original scans of autoradiograms (immunoblots) included in Fig. 6e


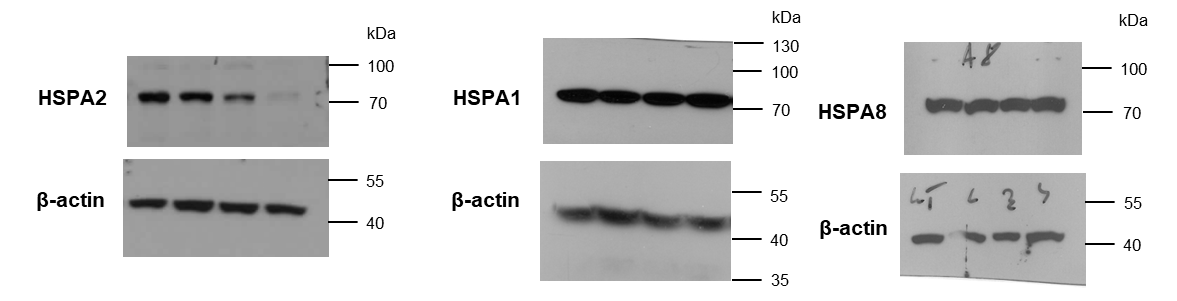


**Figure S11.** Unprocessed original scans of autoradiograms (immunoblots) included in Fig. S1a


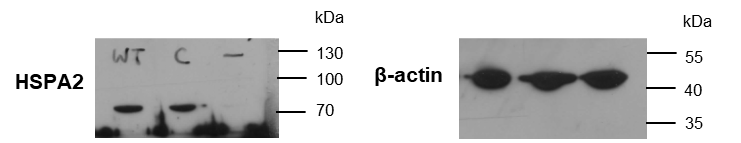


**Figure S12.** Unprocessed original scans of autoradiograms (immunoblots) included in Fig. S2a


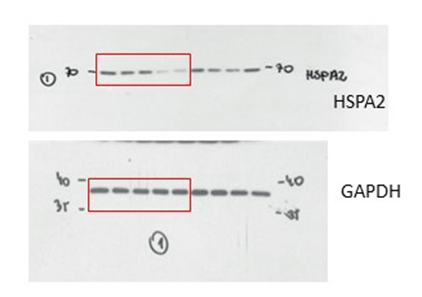


**Figure S13.** Unprocessed original scans of autoradiograms (immunoblots) included in Fig. S4a
